# Profiling of adhesive-related genes in the freshwater cnidarian *Hydra magnipapillata* by transcriptomics and proteomics

**DOI:** 10.1080/08927014.2016.1233325

**Published:** 2016-09-23

**Authors:** Marcelo Rodrigues, Thomas Ostermann, Leopold Kremeser, Herbert Lindner, Christian Beisel, Eugene Berezikov, Bert Hobmayer, Peter Ladurner

**Affiliations:** ^a^ Institute of Zoology and Center for Molecular Biosciences Innsbruck, University of Innsbruck, Innsbruck, Austria; ^b^ Division of Clinical Biochemistry, Biocenter, Innsbruck Medical University, Innsbruck, Austria; ^c^ D-BSSE, ETH Zürich, Basel, Switzerland; ^d^ ERIBA, University of Groningen, University Medical Center Groningen, Groningen, The Netherlands

**Keywords:** *Hydra*, basal disc, bioadhesion, transcriptome, differential transcriptome, proteome

## Abstract

The differentiated ectodermal basal disc cells of the freshwater cnidarian *Hydra* secrete proteinaceous glue to temporarily attach themselves to underwater surfaces. Using transcriptome sequencing and a basal disc-specific RNA-seq combined with *in situ* hybridisation a highly specific set of candidate adhesive genes was identified. A *de novo* transcriptome assembly of 55,849 transcripts (>200 bp) was generated using paired-end and single reads from Illumina libraries constructed from different polyp conditions. Differential transcriptomics and spatial gene expression analysis by *in situ* hybridisation allowed the identification of 40 transcripts exclusively expressed in the ectodermal basal disc cells. Comparisons after mass spectrometry analysis of the adhesive secretion showed a total of 21 transcripts to be basal disc specific and eventually secreted through basal disc cells. This is the first study to survey adhesion-related genes in *Hydra*. The candidate list presented in this study provides a platform for unravelling the molecular mechanism of underwater adhesion of *Hydra.*

## Introduction

Biological adhesion (bioadhesion) is the attachment of organisms to either biotic or abiotic surfaces (Gorb 2008). This capacity occurs from microscopic organisms, such as bacteria, to much larger and more complex marine algae, invertebrates, and terrestrial vertebrates (Smith & Callow 2006; von Byern & Grunwald 2010). Information on how animals solve problems of adherence in diverse environments offers models for bio-inspired technology with applicability in biomedicine (Khanlari & Dubé 2013). Despite years of research in the field, only a few model systems have inspired the development of biomimetic adhesives, eg the case of geckos for dry bioadhesion (Hawkes et al. 2014) and mussels for glue-based attachment (Lee et al. 2007; Westwood et al. 2007). Within glue-based adhesion, the mode of action is better understood in marine than in freshwater organisms. One representative model for freshwater bioadhesion is the caddisfly larvae (order Trichoptera). These larvae produce adhesive silk fibres to construct shelters underwater by gluing material gathered from the environment using a highly phosphorylated adhesive protein (Stewart & Wang 2010). Knowledge of the underlying gene sequences is essential for modelling bioinspired adhesives (Endrizzi & Stewart 2009; Guerette et al. 2013) and often represents the bottleneck of many research projects (Rodrigues et al. 2014).

Transcriptome-based approaches (commonly referred to as RNA-seq) aimed at elucidating the full collection of RNA molecules expressed in an organism, a tissue or a certain cell type have become an important tool for bioadhesion research and hold a promising future in the bioadhesion field (Rodrigues et al. 2014; Hennebert et al. 2015). For example, RNA-seq facilitated the discovery of neuropeptides and peptide hormone involved in barnacle larval settlement (Yan et al. 2012). While transcriptome sequencing retrieves the full complement of mRNA present in the sample, additional quantitative information is necessary to identify transcripts that are highly expressed in the region specific to the adhesive tissue of interest. Differential transcriptomics permit the *in silico* subtraction of different samples yielding a quantitative ranking of differentially expressed transcripts. Based on a region-specific transcriptome of the flatworm *Macrostomum lignano* (Arbore et al. 2015), an adhesion-related intermediate filament gene has been identified (Lengerer et al. 2014) and potential glue candidates are currently under study (Ladurner, unpublished). Thus far, differential transcriptomic studies are becoming popular in bioadhesion research, primarily to identify candidate adhesive genes for reliable downstream analysis. In barnacles, RNA-seq has been applied to compare transcriptomes of the prosoma (non-adhesive tissue) and the basis (which contains the cement gland) of the species *Tretraclita japonica formosana* (Lin et al. 2014). The analyses showed that homologues of barnacle cement proteins and other possible adhesion related-genes, like custrin and fibroin, were predominately expressed at the basis (region responsible for adhesion).

The combination of transcriptomics and proteomics has proven to be a powerful method for directly correlating mRNA and secreted adhesive proteins. Guerette et al. (2013) used tissue-specific transcriptome as a database for peptide prediction after tandem mass spectrometry analysis to identify proteins involved in the adhesive mechanism in the green mussel *Perna viridis*. In the caddisfly larvae *Hesperophylax* sp., the silk gland-specific transcriptome was used to identify homologous H-fibroin contigs, an important component of proteins present in silk fibres (Ashton et al. 2013; Wang et al. 2014). It was also used to identify a novel 114 kDa cement protein in the barnacle *Amphibalanus amphitrite* (Wang et al. 2015), and successfully implemented by Hennebert et al. (2015) for identification of proteins secreted by the tube feet of the sea star *Asterias rubens*. In the scallop *Chlamys farreri* a comparison between several tissue specific transcriptomes and proteomes helped in identifying seven foot-specific proteins, which might be involved in adhesion (Miao et al. 2015). These studies have provided important insights into the sequences of the proteins composing natural adhesives and provide the foundation for the molecular designing of novel biomimetic adhesives.

**Figure 1.**
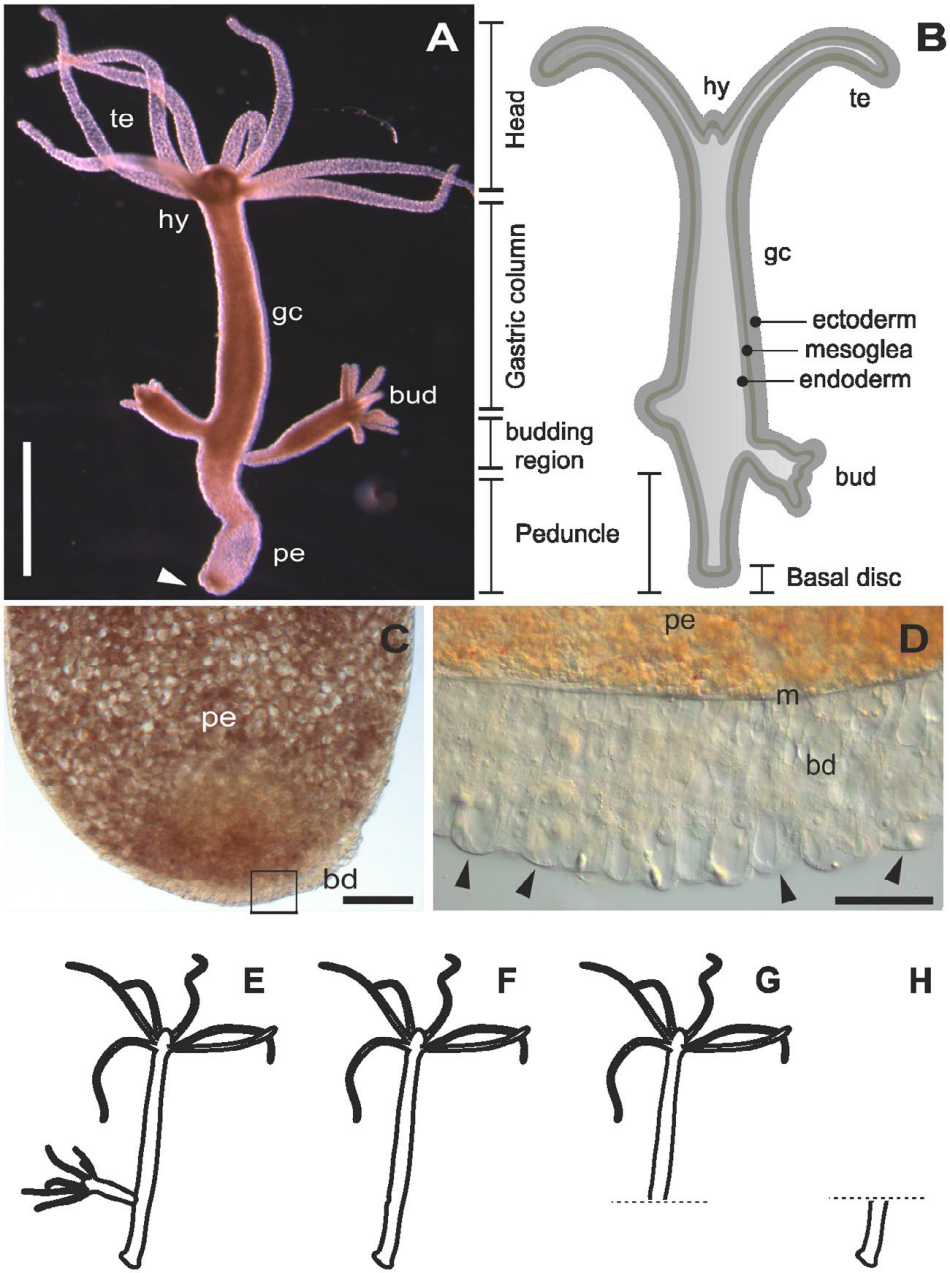
The model organism *Hydra*. (A) Micrograph of an adult polyp. The arrow indicates the basal disc. (B) Scheme of an adult polyp indicating details of the animal morphology. (C) Squeezed preparation of the peduncle region. The square indicates the area magnified in D. (D) The arrowheads point at individual ectodermal basal disc cells. (E–H) Different polyp conditions used for the RNA sequencing, transcriptome assembly and differential gene expression. (E) Whole polyp with bud; (F) whole polyp without bud; (G) amputated anterior part; and (H) amputated peduncle. te=tentacles, hy=hypostome, gc=gastric column, pe=peduncle, m=mesoglea, bd=basal disc. Scale bars=(A) 1 mm, (C, D) 50 μm.

The freshwater cnidarian *Hydra* is a solitary polyp considered to be a member of the most ancient multicellular animal clades in metazoan evolution to develop specialised tissues (Figure 1A and B). They are common inhabitants in any shallow freshwater ponds, lakes and creeks all year round. During its entire life cycle, *Hydra* is able to attach and detach repetitively by establishing effective and reliable underwater adhesion onto the substratum. The animal has a single axis with radial symmetry, which contains a head, body column, and peduncle along the axis. The head consists of two parts: the hypostome in the apex, and the tentacle zone from which the tentacles emerge in the basal part of the head. The body column has three parts: the gastric column in the apical part, peduncle in the basal part, and a budding zone between the gastric column and peduncle. The bud, *Hydra*’s mode of asexual reproduction, emerges from the budding zone. Before detaching from the mother polyp, buds contain the same cellular characteristics of an adult asexual polyp including the modified ectodermal basal disc cells with the ability to secrete adhesives (Hobmayer et al. 2012; Steele 2002). This ability is attained by adhesive secretions that are mainly composed of proteins which are produced and released by only one cell type: basal disc-specific, differentiated ectodermal cells (Figure 1C and D). These ectodermal basal disc cells are characterised by the presence of granules presumably containing the adhesive (Chaet 1965; Philpott et al. 1966; Davis 1973; Rodrigues et al. Forthcoming). Morphologically, four types of granules can be recognised, but their degree of contribution to the secreted adhesive is still not known. Glycan was found to be a constituent of all the granules but with different distributions. Muscle mediated detachment seems to be an important mechanistic component facilitated by basal disc cells Rodrigues et al. (Forthcoming); until now no evidence for chemical detachment was reported in *Hydra*.

Little information is currently available regarding the molecular basis of the bioadhesive of *Hydra*. The ectodermal basal disc cells are known to have peroxidase activity which is related to some granules and neighbouring cytoplasm (Dübel et al. 1987; Hoffmeister-Ullerich et al. 2002; Rodrigues et al. Forthcoming). These peroxidase genes were isolated and sequenced (Hoffmeister-Ullerich et al. 2002; Habetha & Bosch 2005; Thomsen & Bosch 2006). The morphogenetic cell movements which give rise to the formation of peduncle and the basal disc are well known (Dübel et al. 1987; Steele 2002; Hobmayer et al. 2012). However, little molecular information exists about the peduncle, and most studies are related to pattern formation and regeneration. Two proteins involved in patterning are the receptor protein tyrosine kinase gene *shin guard*, which is expressed in the ectoderm of peduncle region (Bridge et al. 2000), and *CnNK*-*2* which is expressed in the endoderm, mainly in the peduncle region (Grens et al. 1996). Moreover, two antibodies, namely AE03 (Amano et al. 1997) and 3G11 (Shirokova et al. 2009), were generated by homogenates of *Hydra* cells, staining both granules and the secretion of basal disc cells (Amano et al. 1997; Shirokova et al. 2009; Rodrigues et al. Forthcoming), but their epitopes and exact subcellular localisation are currently unknown.

The goal of the research described here was to identify genes potentially involved in the adhesive mechanism of *Hydra*. By comparing intact and peduncle-amputated polyps through differential transcriptomics, a list of basal disc-specific candidates was identified. This list was further screened by means of *in situ* hybridisation to track down the transcripts which were exclusively expressed in basal disc cells. ESI-LC-MS/MS was used to confirm that some of the proteins coded by the transcripts are effectively present in the adhesive secretion. This information is important for our understanding of the *Hydra* adhesion molecular mechanism and represents valuable resources for producing synthetic adhesives.

## Materials and methods

### Animal culture

*Hydra magnipapillata* strain 105 was used for all the experiments carried out in this study in compliance with animal welfare laws and policies (Austrian Law for animal experiments, TVG 2012, §1). Mass cultures were kept at 18°C in a hydra culture medium (1 mM NaCl, 1 mM CaCl_2_, 0.1 mM KCl, 0.1 mM MgSO_4_, 1 mM tris-(hydroxymethyl)-amino-methane; pH 7.4 adjusted with HCl). Polyps were fed five times per week with freshly hatched *Artemia* nauplii as previously described (Hobmayer et al. 1997). Under these conditions, animals remained asexual and reproduced by budding. Animals were starved for 24 h before experiments.

### Tissue collection for RNA sequencing

Tissue and RNA preparations were divided into two categories based on the ultimate use of the samples: transcriptome assembly and differential analysis. Tissue for transcriptome assembly was collected from 15 whole polyps containing at least one bud (ie sample ‘Whole hydra’, Figure 1E). Samples for differential analysis were composed by batches of 15 whole polyps without bud (sample ‘Whole polyp’, Figure 1F), 20 polyps with amputated peduncle (sample ‘Anterior part’, Figure 1G), and 50 amputated peduncles (sample ‘Peduncle’, Figure 1H). Three biological replicates were generated for each sample condition. The tissue samples were immediately dissolved in TRI Reagent^®^ (Sigma-Aldrich, Vienna, Austria) for RNA extraction. DNA was removed by extraction with chloroform, and RNA was precipitated in isopropanol, washed with 70% EtOH, and air dried previous to RNA resuspension with RNAse-free water. Quantity and quality (purity and integrity) of purified RNA was assessed by three methods: first, by gel electrophoresis, second by the absorbance at different wavelengths with a NanoDrop 2000c spectrophotometer (Thermo Fisher Scientific, Bremen, Germany), and finally by an Agilent 2100 Bioanalyzer before library preparation (Santa Clara, CA, USA).

To obtain a high-quality transcriptome, state-of-the-art Illumina sequencing for generating three different libraries were used. For the full transcriptome of whole *Hydra*, two Illumina HiSeq 100 bp paired-end using the TruSeq PE Cluster kit v3, and one 250 bp MiSeq paired-end using the HiSeq PE Cluster generation kit. For the differential transcriptome, single reads of HiSeq 50 bp libraries were prepared by using the TruSeq SBS kit v3 for Whole polyp, Anterior part, and Peduncle. The libraries were sequenced with 51 cycles on an Illumina Genome Analyzer II following the manufacturer’s protocol (San Diego, CA, USA).

### Transcriptome assembly, annotation and differential analysis

Illumina reads were assembled separately by two *de novo* transcriptome assembly programs, Trinity v. r2013_08_14 (Grabherr et al. 2011) and IDBA-Tran v. 1.1.1 (Peng et al. 2013). The resulting assemblies were merged with Minimus2 assembler from Amos package v.3.1.0 (Treangen et al. 2011), and transcripts with more than 99% identity over their full length were reduced to single longest transcripts to remove redundancy. Next, transcripts were clustered using cd-hit-est software v.4.5.4 (Li & Godzik 2006) to assign gene groups to alternative transcripts.

For functional domain annotation, transcripts were translated in all six frames using a bioperl script, and the resulting amino acid sequences were searched by hmmer software v. 3.1 (Eddy 2011) against Pfam database v.27 (Finn et al. 2016). Best hits against human proteins and UniRef90 database (Suzek et al. 2007) were annotated with blastx program from BLAST v 2.2.26 (Altschul et al. 1990). The raw reads and resulting transcriptome assembly HYRNA140215 were submitted to NCBI databases and are associated to the BioProject PRJNA324754.

For differential gene expression analysis, Illumina reads were mapped to the transcriptome assembly by Bowtie v.1.0.0 (Langmead et al. 2009) with parameters ‘–best –strata’, and transcript abundancies were calculated using transcripts per million (TPM) metric implemented in Rsem v 1.2.7 (Li & Dewey 2011). Differential gene expression analysis was performed with EBSeq (Leng et al. 2013) through wrapper scripts provided in the Rsem package. The transcriptome assembly completeness was assessed with BUSCO (Simão et al. 2015).

### Preparation for mass spectrometry

Samples were collected from whole intact polyps in triplicates. 15 polyps were placed into a 500 μl low binding-protein tubes and were allowed to secrete material for 1 h at room temperature. Secreted proteins in the 500 μl sample vials were reduced with 50 μl of 10 mM dithiothreitol (solved in 100 mM NH_4_HCO_3_ buffer pH 8) at 56°C for 30 min, and alkylated with 50 μl of 55 mM iodoacetamide (solved in 100 mM NH_4_HCO_3_ buffer pH 8) at RT for 20 min. Samples were digested by adding 1 μg of trypsin (Trypsin gold, Promega, Mannheim, Germany) in 100 mM NH_4_HCO_3_ buffer pH 8, and incubated at 37°C overnight. The tryptic peptides were purified by ZipTip C18 pipette tips (Millipore, Vienna, Austria) according to the manufacturer’s instructions prior to nanoLC-ESI-MS analysis.

Samples were analysed using an UltiMate 3000 nano-HPLC system coupled to a Q Exactive Plus mass spectrometer (both Thermo Scientific, Bremen, Germany) equipped with a Nanospray Flex ionisation source. The peptides were separated on a homemade fritless fused-silica microcapillary column (75 μm i.d. × 280 μm o.d. × 10 cm length) packed with 3 μm reversed-phase C18 material (Reprosil). Solvent for HPLC were 0.1% formic acid (solvent A) and 0.1% formic acid in 85% acetonitrile (solvent B). The gradient profile was as follows: 0–2 min, 4% B; 2–55 min, 4–50% B; 55–60 min, 50–100% B, and 60–65 min, 100% B. The flow rate was 250 ml min^−1.^

### Mass spectrometry data analysis

The Q Exactive Plus mass spectrometer was operated in the data dependent mode selecting the top 12 most abundant isotope patterns with charge > 1 from the survey scan with an isolation window of 1.6 mass-to-charge ratio (m/z). Survey full scan MS spectra were acquired from 300 to 1,750 m/z at a resolution of 70,000 with a maximum injection time (IT) of 120 ms, and automatic gain control (AGC) target 1e6. The selected isotope patterns were fragmented by higher-energy collisional dissociation (HCD) with normalised collision energy of 28 at a resolution of 35,000 with a maximum IT of 120 ms, and AGC target 5e5.

Data analysis was performed using a Proteome Discoverer 1.4.1.14 (Thermo Scientific) with the search engine Sequest. The raw files were searched against the *Hydra* transcriptome generated in the present study (HYRNA140215) which was translated into proteins using transeq pipeline implemented in Galaxy software (Blankenberg et al. 2007). This allowed for 137,663 entries. Precursor and fragment mass tolerance was set to 10 ppm and 0.02 Da, respectively, and up to two missed cleavages were allowed. Carbamidomethylation of cysteine, and oxidation of methionine were set as variable modifications. Peptide identifications were filtered at 1% false discovery rate. Raw data and results were deposited to the ProteomeXchange consortium *via* the Pride (Vizcaíno et al. 2016) partner repository with dataset identifier PXD004899.

### In situ hybridisation screening

Candidate transcripts were selected for *in situ* hybridisation (ISH) screen from the differential transcriptome analysis. As an additional parameter for quality control of the differential expression analysis we included 90 transcripts that were predicted to be differentially expressed in the peduncle (Whole polyp *vs* Anterior part), and the top-10 transcripts predicted to be differentially expressed in the anterior part (Whole polyp *vs* Peduncle). Forward and reverse primer pairs were designed for each candidate transcript with Primer3 version 4.0.0 (Untergasser et al. 2012). A T7-promoter sequence was added to the reverse primer. cDNA was synthesised using peqGold cDNA synthesis kit H Plus (Peqlab) from total RNA extracted from *Hydra* polyps containing at least one bud collected from a mass culture. cDNA templates were amplified with transcript specific primer pairs under the following PCR conditions: 98°C 30 s, (98°C 10 s, between 56°C and 66°C 30 s, 72°C 30 s)×35, 72°C 2 min. PCR products were purified using Wizard SV Gel and PCR Clean-Up System (Promega) and used as template to synthesise single stranded anti-sense Digoxigenin-labelled RNA probes with the DIG RNA Labelling KIT (Roche, Mannheim, Germany). Transcription was performed following the manufacturer’s instructions except for the use of DIG-labelling mixture from Roche, where 1 μl was used per reaction. Two μl of each transcription were checked by gel electrophoresis and quantified using NanoDrop 2000c. Transcription reactions were pre-diluted in hybridisation buffer to a concentration of 5 ng μl^−1^ and conserved at –80°C. The full protocol for the whole mount ISH is available in Table S1 of the Supplemental material. ISH was modified after Grens et al. (1996) with the following changes: (1) about 360 *H. magnipapillata* polyps were pooled together into a 50 ml tube up to the hybridisation buffer step; (2) the buffer used was PBS; (3) for probe hybridisation, 10–15 polyps were transferred from the 50 ml tube to a 1.5 ml Eppendorf tube, placed into an incubator with 55°C and shaking at 350 rpm for 48 h, probe concentration during hybridisation was 0.2 ng μl^−1^; and (4) the steps after hybridisation were slightly modified after Pfister et al. (2007). Animals were transferred from the reaction tubes into 24 mesh baskets (53 μm mesh size; INTAVIS Bioanalytical Instruments AG, Germany), which were placed into custom made holes drilled into the lid of a 24-well tissue culture plate (Figure S1A–B). Heating steps after hybridisation were performed in a water bath. Plates with pre-warmed buffers were prepared and the lid with the baskets and animals was transferred to the successive solution. For colour development, animals were moved to a 24-well plate without baskets. The colour development was at 4°C for ensuring a slow reaction using the NBT/BCIP system (Roche). Processed specimens were mounted in Gevatol medium. For bright field or differential interference contrast visualisation, samples were examined with a Leica DM5000, Vienna, Austria. Images were taken with a Leica DFC495 digital camera and Leica LAS software.

## Results

### An H. magnipapillata strain 105 specific transcriptome

At the beginning of the present study, no transcriptome of *H. magnipapillata* was publicly available. Therefore, a transcript sequence database was generated from both Illumina paired-end and single reads by *de novo* transcript assembly.

To generate a high quality transcriptome for downstream analysis, a total of 9,648,108 raw reads passing Illumina filter were generated by 250 bp MiSeq paired-end sequencing, 27,631,126 reads by 100 bp HiSeq paired-end sequencing, and 58,941,681 50 bp single reads. *De novo* transcriptome assembly of *H. magnipapillata* was executed by combining all the reads from all different sequencing strategies. After removing transcripts shorter than 200 bp in length, the final transcriptome assembly accounted for 55,849 transcripts, with a N50 of 1,672 bp in length. The statistics of these data are summarised in Table 1. This Transcriptome Shotgun Assembly project has been deposited at DDBJ/EMBL/GenBank under the accession GEVZ00000000. The version described in this paper is the first version, GEVZ01000000.

**Table 1.** Summary of the *Hydra magnipapillata* transcriptome assembly.

Total number of transcripts	55,849
Total length (bp)	57,061,933
Longest transcript (bp)	31,041
Shortest transcript (bp)	201
Average transcript length (bp)	1,022
N50 length (bp)	1,672

A published dataset containing 843 metazoan orthologues was used to assess the assembled *Hydra* transcriptome completeness with BUSCO (Simão et al. 2015). With BUSCO analysis, orthologues are considered to be complete (C) if the length of their aligned sequence is within two standard deviations of the BUSCO group’s mean length, otherwise they are classified as fragmented (F). Complete genes found with more than one copy are classified as duplicated (D), and are classified as missing (M) when the expected score is not met. The analysis resulted in the following BUSCO notation: C:87% [D:26%], F:3.0%, M:9.0%, n:843. This confirms that the generated *H. magnipapillata* transcriptome is of high quality and is a valuable source for downstream applications such as differential gene expression analysis.

### A peduncle-specific differential transcriptome

**Figure 2.**
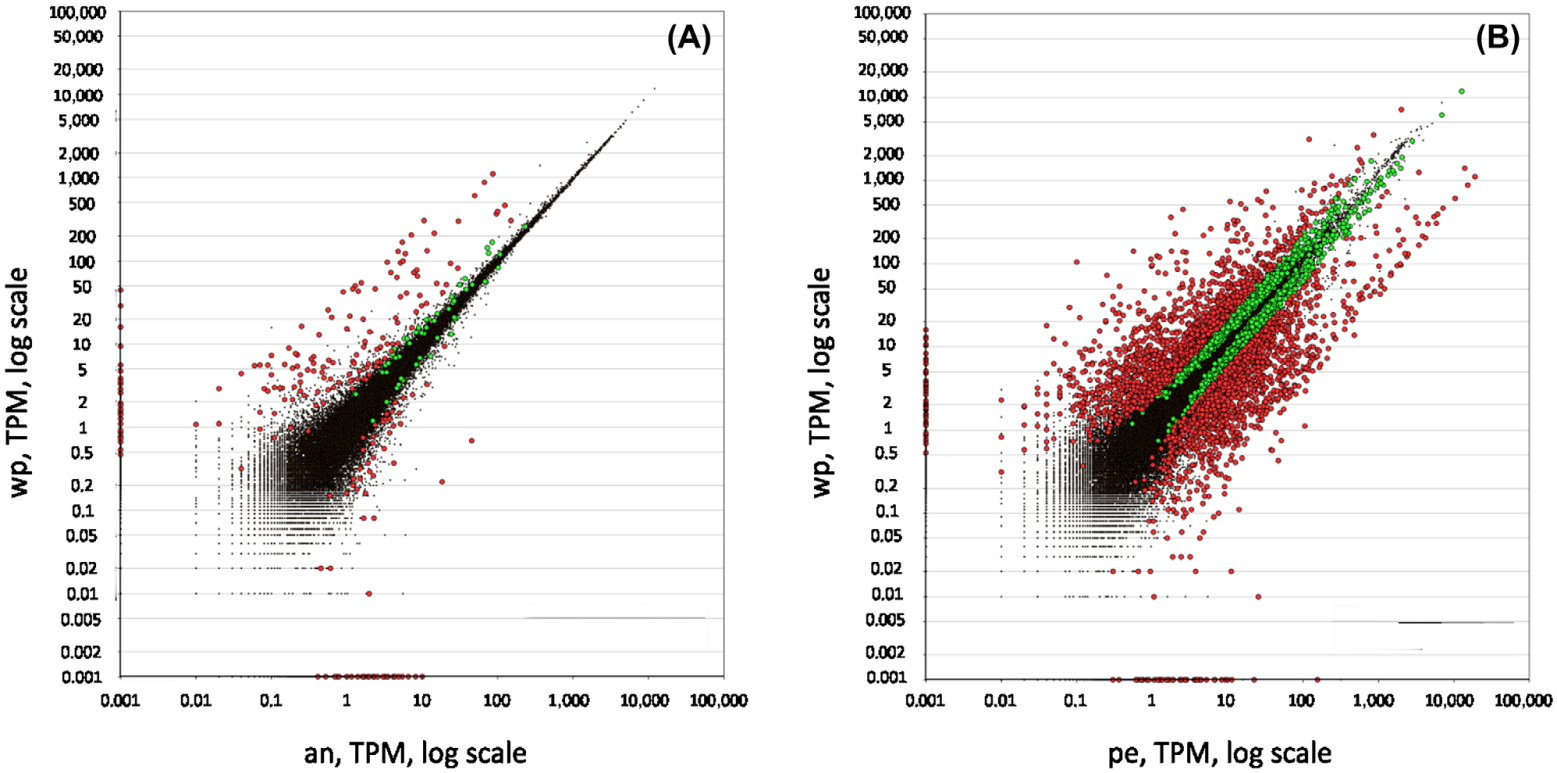
Distribution of expression values between (A) the whole polyp *vs* the anterior part and (B) the whole polyp *vs* the peduncle. Red dots symbolise transcripts with fold change equal or higher than 2, green dots represent fold change equal or smaller than 2, and black dots symbolise transcripts not passing the threshold of 0.95 for the estimated posterior probability of differential expression. wp: whole polyp, an=anterior part, pe=peduncle.

To generate a peduncle-specific candidate list, RNA-seq technology was used to compare transcriptomic profiles of intact polyps without buds (Figure 1F) and polyps with amputated peduncle (Figure 1G), which lack differentiated ectodermal basal disc cells. To determine differentially expressed transcripts in the peduncle, the reads from the control sample Whole polyp (46,192,895 raw reads passing Illumina filter, 50 bp reads) and reads of Anterior part (46,809,132 50 bp reads) were mapped against the generated *H. magnipapillata* transcriptome. The differential expression analysis retrieved a raw list of 279 transcripts with Posterior Probability Differentially Expressed (PPDE) ≥0.95, of which 226 transcripts showed a ≥ twofold change in the peduncle (Figure 2A). This collection of 226 transcripts constitutes the peduncle specific transcript candidates. To generate a highly peduncle-specific candidate list, the raw list was filtered for transcripts with the abundance of more than five transcripts per million (TPM) in Whole polyp, and sorted from higher to lower LOG2 fold change (Table S2). The rational to consider only transcripts with more than 5 TPM is based on the observation that vesicles containing potential glue proteins are only present in the basal disc cells, they are abundant and constantly produced within these cells (Rodrigues et al. Forthcoming). Known proteins present in basal disc cell, such as eg. Peroxidase transcripts, accumulate up to 96 TPM. Therefore, 5 TPM was considered as a suitable threshold, and lower expressed transcripts most likely do not represent potential glue proteins.

To further validate that the analysis was unbiased, the ‘peduncle’ sample (Figure 1H; 52,921,184 50 bp reads) was compared to the ‘Whole polyp’ sample, which should yield candidate transcripts expressed exclusively in the anterior part of the animals including tentacles, head, and gastric column. Although the resulting differentially expressed transcripts of Whole polyp *vs* Peduncle are not relevant for the adhesive related transcripts, it is highly informative to ensure the analysis was accurate. The generated anterior part specific raw list rendered 2,679 transcripts with PPDE ≥ 0.95 and ≥ twofold change (Figure 2B, Table S3). A highly anterior part-specific candidate list was generated by filtering the raw candidates for having more than 40 TPM in whole polyp and less than 10 TPM in the peduncle (Table S3). The rational for the selection of 40+TPM is to ensure a high expression in the anterior part. The choice of 10 TPM in the peduncle is based on experience of previous differential expression studies (Ladurner, unpublished). In theory, an anterior-specific transcript should not have mapped reads in the peduncle. However, an anterior-specific transcript might be a member of a larger gene family that exhibit a certain sequence overlap with other transcripts – resulting in cross-mapping of short reads. For example, in *M. lignano*, a tail-specific intermediate filament related transcript involved in adhesion was identified (Lengerer et al. 2014). Intermediate filaments comprise a large protein family with various types and isoforms (eg keratin, desmin, vimentin, lamin, and neurofilaments), which results in a distribution of mapped reads within similar sequence regions. Therefore, a non-stringent number of 10 TPM was chosen for selecting anterior-specific candidates.

In summary, this strategy rendered a highly specific candidate list of transcripts differentially expressed either in the peduncle or in the anterior part. These candidates were the ones selected for ISH screening.

### Spatial gene expression: in situ hybridisation screening

**Figure 3.**
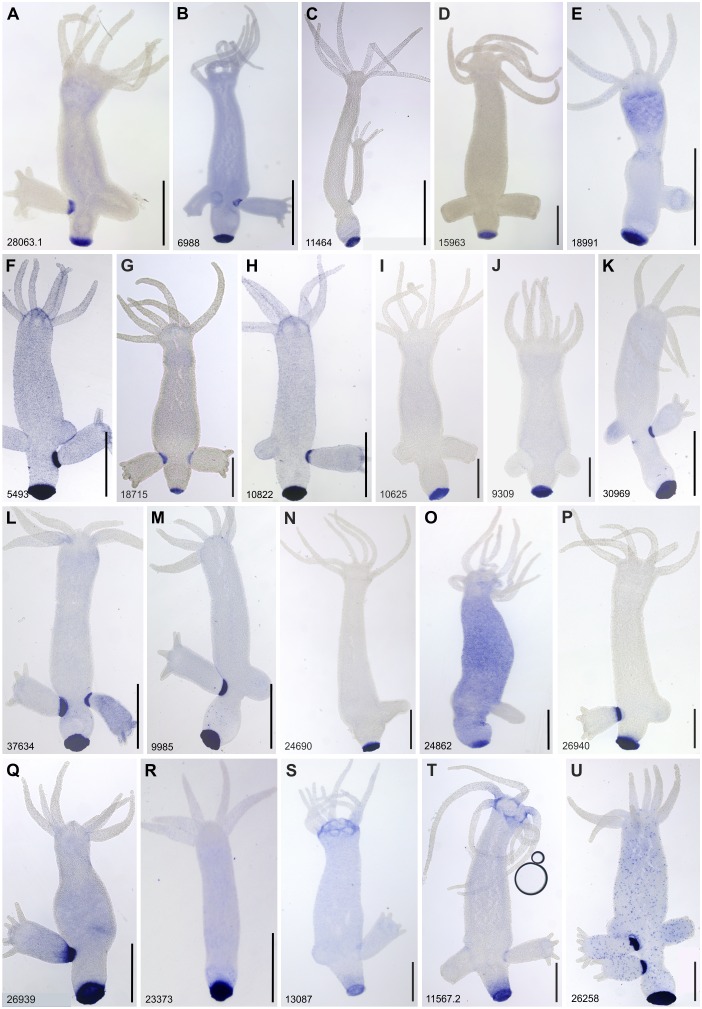
Whole mount *in situ* hybridisation of transcripts expressed in the peduncle and potentially involved in the adhesive mechanism of *Hydra*. Scale bars: 500 μm.

The differential gene expression analysis resulted in 90 transcripts as candidates with potential basal disc specific expression (Table S2). An ISH screen was performed for all candidates. An adapted ISH protocol for *Hydra* polyps was established by using a 24-well plate system (Table S1 and Figure S1). Thirty-five transcripts were exclusively expressed in the basal disc cells (Figure 3A–R; Figure S2A–Q). Two transcripts showed expression in basal disc cells and at the base of tentacles (Figure S3S and T), one had expression in basal disc and in cells of the gastric column (Figure 3U), one was expressed in basal disc, gastric column and hypostome (Figure S3A), and a last one in the endoderm of basal disc and whole body endoderm (Supplementary Information Figure S3B). In addition, the ISH screen revealed expression of four transcripts in interstitial cells in the gastric column (Figure S4A–D), four in the peduncle (Figure S4E–H), one in the peduncle and bud (Figure S4I), one provided a weak ISH signal in the bud, which could be related to bud detachment from mother polyp, but not directly involved in adhesion (Figure S4J), and one in the tentacles (Figure S4K).

To corroborate bioinformatic analysis of candidate transcripts expressed in the anterior part of the animal (Table S3), ISH was performed with eight of the top-10 candidates (Figure S5). Seven out of eight were expressed in the head (Figure S5A–G), and one showed expression in cells at the gastric column (Figure S5H).

### Annotation of basal disc specific genes

Out of 40 transcripts expressed in the basal disc, 22 scored an annotation after Protein BLAST (Table S3). The largest group of annotated proteins comprised molecules with an apparent structural function that are characteristic for extracellular matrix proteins involved in protein–protein and protein–ligand interactions. Protein domain were searched using Protein family (Finn et al. 2016) http://pfam.xfam.org/. The identified motifs include galactose binding lectin, chitin binding peritrophin-A, DOMON, leucine rich repeats, and fibronectin type III. Four transcripts were related to protease inhibitor function including the serine-type endopeptidase inhibitor from the antistasin family, and the cysteine-type cystatin domain. Two transcripts encoded glycosidase hydrolases of the alpha-L-arabinofuranosidase B domain with the ability of breaking linkages in both oligosaccharides and polysaccharides. Two transcripts encoded for the haem peroxidase enzyme. Another two transcripts contained the FRG1-like domain motif, a domain with unknown function. In a BLAST search against UniProt database, the two transcripts annotated with a FRG1-like domain were shown to be similar to PPOD1 (Thomsen & Bosch 2006) commonly present in *Hydra* species. Finally, one protein domain was involved in nitrogen metabolism – glutamine synthetase.

From the 18 transcripts with non-PFam annotation, 12 were predicted in the published *H. magnipapillata* genome as uncharacterised protein, and another as tenascin-X-like, an extracellular matrix glycoprotein. Five transcripts were not predicted in the published *Hydra* genome (Chapman et al. 2010); four of these have homologues to other phyla but did not contain PFam domains, and a last one showed no detectable homologues in the databases (Table S2). The number of novel and structural proteins is markedly pronounced in *Hydra* basal disc, which might indicate a large diversity between adhesive proteins of different taxa or a higher morphological complexity of hydrozoan ectodermal basal disc cells.

In addition to examining basal disc gene expression, the material secreted from *Hydra* was analysed using mass spectrometry to find transcripts that are secreted by ectodermal basal disc cells. The MS/MS results were merged and only the proteins identified in the three replicates were taken into account (Table S4). A total of 21 basal disc specific transcripts matched a protein in the secreted material (Table 2, Figure 3). The transcript HYRNA1402_9309 was identified in the three replicates with only one peptide while the other 20 transcripts were mapped with more than three peptides (Table S4).

**Table 2.** Top Blast hits of the potential adhesive proteins by protein family and the NCBI non-redundant database.

Transcript ID	FC^1^	Protein family	Query length (aa)	NCBI description	Species	Accession number	Identity%	Alignment length (aa)	Hit length (aa)	NCBIE-value	Bit score
HYRNA1402_13087	10,08	FRG1-like family [PF06229.7]	290	Uncharacterised LOC100202041precursor	*Hydra vulgaris*	NP_001296632.1	98	283	290	0.0	559
HYRNA1402_28063.1	5,41	N.A.^3^	141	Hypothetical protein	*Crassostrea gigas*	EKC32775.1	48	59	126	5e-30	114
HYRNA1402_6988	5,17	Alpha-L-arabinofuranosidase B (ABFB) [PF05270.8]	310	alpha-L-arabinofuranosidase B domain^2^	*Hydra vulgaris*	XP_002167824.2	99	284	310	0.0	573
HYRNA1402_11464	5,06	Peroxidase [PF00141.18]	305	Putative ascorbate eroxidase^2^	*H. vulgaris*	XP_002161794.1	99	303	369	0.0	637
HYRNA1402_15963	4,89	N.A.	264	Multiple epidermal growth factor-like domains protein 11^2^	*H.vulgaris*	XP_002166617.1	100	264	264	0.0	542
HYRNA1402_18991	4,84	DOMON domain [PF03351.12]	198	Uncharacterised protein LOC100209978	*H. vulgaris*	XP_004212051.1	99	198	198	5e-119	346
HYRNA1402_5493	4,80	Alpha-L-arabinofuranosidase B (ABFB) [PF05270.8]	588	Uncharacterised protein LOC100202073	*H. vulgaris*	XP_002159690.1	100	588	598	0.0	1,208
HYRNA1402_18715	4,79	Galactose binding lectin domain [PF02140.13]	208	Rhamnose-binding lectin-like^2^	*H. vulgaris*	XP_012565931.1	68	141	405	4e-97	298
HYRNA1402_10822	4,78	Peroxidase [PF00141.18]	365	Putative ascorbate peroxidase^2^	*H. vulgaris*	XP_012561209.1	93	339	348	0.0	693
HYRNA1402_26258	4,71	Galactose binding lectin domain [PF02140.13]	166	Rhamnose-binding lectin-like^2^	*H. vulgaris*	XP_012565931.1	96	160	405	3e-98	297
HYRNA1402_10625	4,32	Galactose binding lectin domain [PF02140.13]	311	Rhamnose-binding lectin-like^2^	*H. vulgaris*	XP_012565931.1	99	307	405	0.0	627
HYRNA1402_9309	4,12	Chitin binding peritrophin-A domain [PF01607.19]	165	Uncharacterised protein LOC100199767	*H. vulgaris*	XP_002165607.1	100	165	173	1e-101	310
HYRNA1402_30969	3,84	N.A.	137	Uncharacterised protein LOC100205981	*H. vulgaris*	XP_012558233.1	100	134	1,421	2e-73	247
HYRNA1402_37634	3,58	N.A.	110	Uncharacterised protein LOC100205981	*H. vulgaris*	XP_012558233.1	100	110	1,421	2e-65	223
HYRNA1402_9985	3,50	N.A.	415	Uncharacterised protein LOC105845407	*H. vulgaris*	XP_012558601.1	81	335	764	0.0	670
HYRNA1402_24690	3,27	N.A.	178	Uncharacterised protein LOC105845407	*H. vulgaris*	XP_012558601.1	77	137	764	3e-89	285
HYRNA1402_24862	3,19	Cystatin domain [PF00031.16]	136	Cystatin JZTX-75-like^2^	*H. vulgaris*	XP_002162922.2	99	135	138	3e-90	268
HYRNA1402_11567.2	2,87	FRG1-like family [PF06229.7]	290	Peroxidase PPOD1-like precursor^2^	*H. vulgaris*	NP_001267756.1	96	277	290	0.0	572
HYRNA1402_26940	1,93	Galactose binding lectin domain [PF02140.13]	131	Rhamnose-binding lectin-like^2^	*H. vulgaris*	XP_002166914.1	89	123	403	1e-73	234
HYRNA1402_26939	1,89	Galactose binding lectin domain [PF02140.13]	131	Rhamnose-binding lectin-like^2^	*H. vulgaris*	XP_002166914.1	100	131	403	5e-86	266
HYRNA1402_23373	1,85	Galactose binding lectin domain [PF02140.13]	157	Rhamnose-binding lectin-like^2^	*H. vulgaris*	XP_002166914.1	100	157	403	1e-104	315

Notes: The list is sorted by log2 fold change after differential expression analysis.

^1^ Transcript fold change (FC) in log2 between Whole polyp and Anterior part samples.

^2^ Annotations are from uncharacterised proteins.

^3^ No protein family was recognisable. Transcripts shaded in light grey are expressed in the basal disc cells only, and those shaded in dark grey are expressed in basal disc and in other cells of the polyp.

## Discussion

The ectodermal basal disc cells of *Hydra* are to the authors’ best knowledge the only studied cell type with the combined ability of secreting adhesive for substratum attachment and simultaneously performing muscle-mediated detachment Rodrigues et al. (Forthcoming). The Illumina sequencing effort used in the present work led to a reliable transcriptome assembly and differential gene expression results. While undertaking the present work, two transcriptomes for the *H. magnipapillata* 105 strain were published from different sources (Juliano et al. 2014; Petersen et al. 2015). There is a considerable discrepancy in the number of assembled transcripts between the different works (27,128 transcripts, Juliano et al. (2014); 36,338 transcripts, Petersen et al. (2015); and 55,849 transcripts, present work). The use of different strategies for the transcriptome assembly is a hindrance for a more detailed comparison. For example, Petersen et al. (2015) set a number of 400 bp as cut-off for their assembly while the present work set 200 bp. In another *Hydra* strain (*Hydra vulgaris* strain Basel), RNAseq transcriptome yielded 48,909 assembled transcripts (Wenger & Galliot 2013). In addition, the first differential transcriptome dataset for the peduncle region is presented, which combined with the footprint proteomic data provides first candidates for molecules involved in the temporary adhesive mechanism of *Hydra*. The expression survey produced a library of 21 candidates for looking more deeply into the molecular mechanism of temporary adhesion in *Hydra*. Therefore, these results provide critical information to further explore individual gene function in the underwater adhesive process.

The main constituent of the adhesive secreted by the ectodermal basal disc cells of *Hydra* are proteins (Rodrigues et al. Forthcoming). One of the transcripts expressed in the basal disc codes for the enzyme glutamine synthetase. It acts as a key enzyme controlling the use of nitrogen inside cells to build nitrogen-rich molecules, such as proteins (Eisenberg et al. 2000). The high expression of this enzyme in the basal disc can be related to the fact that ectodermal basal disc cells must produce many copies of the genes coding for adhesive proteins in order to meet the demands of reversible adhesion. The fact that marine organisms need to produce many copies of the adhesive proteins to meet the demands of bioadhesion has been previously mentioned for mussels (Warner & Waite 1999) and for sea urchins (Lebesgue et al. 2016). For the present experiments the polyps were detached from the substratum before amputation and RNA extraction. This procedure could have triggered the biosynthesis of new adhesive proteins. It would be interesting to perform new experiments without detachment to elucidate whether the levels of glutamine synthetase are affected.

Some of the annotated adhesive-candidate transcripts are possibly involved in the *Hydra* adhesive mechanism. Notably, eight transcripts were annotated with glycan-binding functions, suggesting that glycans can be an important component of the adhesive. Post-translational modifications (PTM) such as glycosylation and phosphorylation are common features of several adhesive proteins (Sagert et al. 2006; Flammang et al. 2009; Stewart et al. 2011). The presence of glycans in the adhesive secretory granules of *Hydra* has been reported Rodrigues et al. (Forthcoming). Several adhesive proteins performing either permanent or temporary adhesion have been found to be glycosylated in other systems, such as in the marine sea stars, barnacles and green mussels (Ohkawa et al. 2004; Zhao et al. 2009; Hennebert et al. 2010; Kamino et al. 2012), in freshwater zebra mussels (Rzepecki & Waite 1993), and also in the stick threads secreted by terrestrial velvet worms (Graham et al. 2013). Both N- and O-glycosylation were reported, as is the case of N-glycosylation of cp52 k in barnacles, and O-linked glycans primarily to threonine residues in green and zebra mussels Pvfp-1 and Dpfp-1 (Rzepecki & Waite 1993; Ohkawa et al. 2004; Zhao et al. 2009). They were also reported for C-mannosylated tryptophan, and C-mannosylated hydroxyl-tryptophan (Ohkawa et al. 2004; Zhao et al. 2009). The sea star adhesive was proposed to contain at least two glycoproteins, Sfp-290 which contains mainly N-linked oligosaccharides, and Sfp-210 containing mainly O-linked oligosaccharides (Hennebert et al. 2010). To some extent, the proteins present in *Hydra* adhesive could be O-glycosylated given the presence of the enzyme glycosyl hydrolase AbfB (present results) which is known to hydrolase O-glycosyl compounds.

Transcripts with homology to galactose-binding lectin domain were abundant in the differentially expressed *Hydra* peduncle. The presence of such a domain in the secreted adhesive raises the possibility that non-covalent cross-links contributed by glycan protein binding might be essential to adhesive cohesion in *Hydra*. Non-covalent cross-linking of polysaccharide chains by carbohydrate-binding proteins had been proposed to support the adhesive proteins secreted by terrestrial gastropod (Pawlicki et al. 2004). Lectin staining and proteins with lectin binding domains have been reported in the adhesive secretions of sea stars, sea urchins, frogs, flatworms, and velvet worms (Fleming et al. 2009; Hennebert et al. 2010; Graham et al. 2013; Toubarro et al. 2016; Lengerer et al. 2016). An investigation using lectins to study sea stars adhesive (Hennebert et al. 2010) showed that some glycans, such as galactose, N-acetylglucosamine, fucose and sialic acid residues were linked to the sea star footprint proteins and could provide both cohesive and adhesive properties. The sea urchin adhesive protein Nectin (Nec-1 and Nec-2) also has six tandemly repeated galactose binding domains (Lebesgue et al. 2016; Toubarro et al. 2016), similar to the sea star adhesive protein 1 (Sfp1) which has three of these domains (Hennebert et al. 2014). So, the presence of galactose binding domains in different adhesive systems seems to indicate that it is an important trait in aquatic adhesion. However, treatment of frog’s glue hydrogel with a cocktail of carbohydrate-degrading enzymes did not affect their mechanical integrity. This suggested that glycan-binding interactions did not underpin the structure of frog’s glue (Graham et al. 2013). The lectin binding domains could offer additional properties like resistance to bacterial degradation for the secreted adhesive. They are well known to be part of the constitutive antimicrobial secretions of fish mucus (Thongda et al. 2014), and in combination with a cystatin domain, to protect foam nests for incubating eggs and sperm produced by frog (Fleming et al. 2009). In general, the importance of glycans in the adhesive proteins are still under debate, and therefore, their ultimate role in adhesion remains to be determined.

Additionally, a type of chitin binding domain (chitin-binding peritrophin A) was found to be differentially expressed in basal disc cells (present results), and was also identified in the *Hydra* nematocyte proteome (Balasubramanian et al. 2012), but its function was not further investigated. Chitin (chain polymer of N-acetylglucosamine) is structurally part of hard biomaterials such as squid beaks (Tan et al. 2015), but it is not often associated as part of glue based bioadhesive composites. A surprising association of chitin to glue based adhesion is its involvement with adhesion in marine and freshwater caulobacters (Merker & Smit 1988), and the similarities found to chitin binding protein in the aqueous glue coating the silk fibres of spiders (Choresh et al. 2009). In the adhesive secreted by *Hydra*, this protein domain is likely to bind chitin that would be highly useful in its function to crosslink with other structural adhesive protein(s).

Based on morphological evidence, detachment in *Hydra* was proposed to be driven mainly by mechanical forces (Rodrigues et al. Forthcoming). In other model organisms for bioadhesion using duo-gland systems, it is believed that de-adhesive substances are secreted to facilitate detachment (Flammang et al. 1998; Lengerer et al. 2014). In recent studies several proteases and glycosydases have been identified as being over-expressed in the adhesive discs of sea stars (Hennebert et al. 2015) and sea urchins (Lebesgue et al. 2016), being pointed out as potential components of the de-adhesive secretions. So far, no de-adhesive systems have been described in detail. In the present results, two types of protease inhibitors and one type of glycosylase (glycosyl hydrolase AbfB) were exclusively expressed in the basal disc cells. Given that *Hydra* adhesive is composed of proteins and glycans, a de-adhesive substance could participate in the basal disc detachment. Therefore, the components should have the catalytic ability of degrading protein and break glycan bonds. The detachment mechanism of ectodermal basal disc cells is still poorly characterised, but the present results can be an indication that a combination of mechanical and enzymatic detachment could take place.

In terms of previous known genes in the basal disc, four variants of peroxidase were found. The actual function of peroxidases in *Hydra* are still to be elucidated (Technau et al. 2003; Habetha & Bosch 2005; Thomsen & Bosch 2006). In bioadhesion, peroxidases are known to participate in the catalysis of di-tyrosine formation to post-draw silk fibres produced by the caddisfly larvae (Wang et al. 2014), and in the hardening of egg fertilisation membranes of sea urchin (Foerder & Shapiro 1977). It was also found to be present in the adhesive secretions of other organisms such as sea stars, sea urchins, and barnacles (Hennebert et al. 2015; Wang et al. 2015; Lebesgue et al. 2016). The possible functions of peroxidases in the adhesive secreted by *Hydra* were discussed by Rodrigues et al. (Forthcoming), and are similar to the ideas proposed for sea urchins by Lebesgue et al. (2016). In brief, peroxidase could have multiple functions such as the ability to catalyse the adhesive polymerisation or they could act as antioxidant. Although *Hydra* and sea urchins possess different bioadhesion behaviours, peroxides may act in a similar way in their secreted adhesives.

Taxonomically restricted genes or lineage-speciﬁc genes contribute to morphological diversiﬁcation in metazoans and provide unique functions for particular taxa in adapting to speciﬁc environments. About 40% of the transcripts expressed in the basal disc had no annotated function and even no orthologue in other metazoans. Novel adhesive proteins were discovered for several organisms, including barnacles, one of the most studied systems (Bacchetti De Gregoris et al. 2009; Jonker et al. 2014; Wang et al. 2015). Already stressed by Jonker et al. (2014), more sequence data spanning throughout the metazoan are necessary to identify conserved adhesive domains and gain insight into the relationship between sequence structure and protein function.

In conclusion, this is the first report of integrated differential transcriptomic and mass spectrometry data aiming at discovering the adhesive protein precursors of the freshwater cnidarian *Hydra*. Based on sequences assembled from an Illumina-generated transcriptome dataset the unbiased differential expression enabled the authors to pinpoint matches with secreted proteins. Therefore, transcripts potentially involved in the temporary bioadhesion mechanism of *Hydra* were identified. Functional confirmation of the proteins coded by the basal disc specific transcripts identified in the present study will be essential for the development of an adhesion model mechanism. A better understanding of the *Hydra* adhesive will provide valuable biotechnological resources and guide efforts to develop synthetic adhesives.

## Disclosure statement

No potential conflict of interest was reported by the authors.

## Funding

This work was supported by the Marie-Curie FP7-PEOPLE-2013-IEF [grant 626525]; Austrian Science Fund (FWF) [grant 25404-B25]; COST Action [grant TD0906]; and Förschungsforderungspreis from Hypo Tirol Bank.

## Supplemental data

The supplemental material for this paper is available online at http://dx.doi.org/10.1080/08927014.2016.1233325

## Supplementary Material

GBIF_1233325_Supplementary_material.docx
