# Supplementary material for: Profiling of adhesive-related genes in the freshwater cnidarian Hydra magnipapillata by transcriptomics and proteomics
Source: Biofouling. 2016 Sep 23;32(9):1115–29. doi: 10.1080/08927014.2016.1233325 (PMC5080974; doi:10.1080/08927014.2016.1233325)
Supplement: GBIF_1233325_Supplementary_material.docx [file gbif_a_1233325_sm3470.docx]

**Table S1.** In situ hybridization protocol used for spatial expression experiments.

| **Relaxation** | - Place polyps into tubes |
| --- | --- |
|  | - Remove culture medium, add 4°C cold 2% Urethane in culture medium for 3 min |
|  | - Fix animals by adding 4% PFA in culture medium, incubate at 4°C overnight |
|  | - Remove fixative and wash 4x 5 min Phosphate Buffered Saline 0.1% tween (PBSw) |
|  | - 5 min 25% methanol / 75% PBSw |
|  | - 5 min 50% methanol / 50% PBSw |
|  | - 5 min 75% methanol / 25% PBSw |
|  | - 2x 5 min 100% methanol |
| **Rehydration** | - 5 min 75% methanol / 25% PBSw |
|  | - 5 min 50% methanol / 25% PBSw |
|  | - 5 min 25% methanol / 75% PBSw |
|  | - 2x 5 min PBSw |
| **Proteinase** | - 10 min Proteinase K (20 µg/ml in PBSw) |
|  | - 2x 5 min Glycine (4mg/ml in PBSw) |
|  | - 3x 5 min PBSw |
|  | - 2x 5 min 0.1% Triethanolamine diluted in DEPc water |
|  | - Premix 0.1% Triethanolamine with Acetic Acid (400 : 1) and incubate for 5 min |
|  | - Repeat previous step but now with 200 : 1 |
|  | - 2x 5 min PBSw |
| **Re-fixation** | - 20 min 4% PFA in PBS |
|  | - wash 4x 5 min PBSw |
|  | - heat fixation at 80°C for 20 min in PBSw |
| **Pre-hybridization** | - 10 min 50% hybridization buffer / 50% PBSw |
|  | - 2x 5 min hybridization buffer |
|  | - in a thermos shaker, incubate samples for minimum 2h at 55 °C with 350 RPM |
| **Probe denaturation (parallel steps)** | - In a 1.5 ml tube add hybridization probe to 0.2 ng/µl final concentration in hybridization buffer for 5 min at 95°C |
|  | - Gently remove hybridization buffer from specimens |
|  | - Snap chill on ice denaturated probe and add to tube containing specimens |
| **Hybridization** | - Incubate for 48 h at 55 °C with 350 RPM |
| **Hybridization washes** | - 5 min 100% hybridization buffer at 62°C |
|  | - 5 min 75% hybridization buffer / 25% 2x SSC at 62°C |
|  | - 5 min 50% hybridization buffer / 50% 2x SSC at 62°C |
|  | - 5 min 25% hybridization buffer / 75% 2x SSC at 62°C |
|  | - 2x 30 min 2x SSC containing 0.1% CHAPS at 62°C |
| **Antibody** | - wash 2x 10 min with Maleic Acid Buffer at RT |
|  | - 1 h blocking buffer at 4°C |
|  | - overnight at 4°C with Anti-DIG Alkaline Phosphatase in blocking buffer (1:200) |

**Continuation Table S1**

|  | - wash 7x 10 min with Maleic Acid Buffer |
| --- | --- |
| **Colour development** | - 2x 5 min with Alkaline Phosphatase buffer (NTMT) |
|  | - Develop reaction in NTMT with NBT/BCIP (1 : 50) at 4°C |
|  | - To stop reaction add 100% ethanol and change twice |
|  | - Wash 2x 15 min with PBSw |
|  | - Mount in gevatol medium |

**Table S2.** Peduncle-specific candidate list transcripts screened by in situ hybridization. Transcripts shaded in light grey are expressed in basal disc cells only, and shaded in dark grey are expressed in basal disc and other cells in the polyp. N.A.= no annotation.

| **Transcript id** | **In Situ Hybridization Pattern** | **LOG2 Fold Change** | **Protein Family [Pfam code]** |
| --- | --- | --- | --- |
| HYRNA1402_13087 | Basal disc and base of tentacles | 10,08 | FRG1-like family [PF06229.7] |
| HYRNA1402_6667 | Tentacles | 8,82 | Intermediate filament protein [PF00038.16 9] |
| HYRNA1402_21330.2 | Unspecific | 7,73 | Domain of unknown function (DUF4499) [PF14934.1] |
| HYRNA1402_9725 | Unspecific | 7,57 | Inhibitor of apoptosis-promoting Bax1 [ PF01027.15] |
| HYRNA1402_28063.2 | Basal disc | 6,87 | N.A. |
| HYRNA1402_166 | Unspecific | 6,10 | HECT-domain (ubiquitin-transferase) [PF00632.20] |
| HYRNA1402_5401 | Primer design failed | 5,85 | N.A. |
| HYRNA1402_10161.2 | Unspecific | 5,81 | Snf7 [PF03357.16] |
| HYRNA1402_7286 | Unspecific | 5,49 | Common central domain of tyrosinase [PF00264.15] |
| HYRNA1402_28063.1 | Basal disc | 5,41 | N.A. |
| HYRNA1402_20914.1 | Unspecific | 5,41 | N.A. |
| HYRNA1402_18040 | unspecific | 5,28 | N.A. |
| HYRNA1402_6988 | Basal disc | 5,17 | Alpha-L-arabinofuranosidase B (ABFB) [PF05270.8] |
| HYRNA1402_22666 | Basal disc | 5,14 |  |
| HYRNA1402_11464 | Basal disc | 5,06 | Peroxidase [PF00141.18] |
| HYRNA1402_5980 | Unspecific | 5,06 | Chitin binding Peritrophin-A domain[PF01607.19] |
| HYRNA1402_13531 | Unspecific | 4,91 | N.A. |
| HYRNA1402_15963 | Basal disc | 4,89 | N.A. |
| HYRNA1402_18991 | Basal disc | 4,84 | DOMON domain [PF03351.12] |
| HYRNA1402_13209.1 | Bud | 4,83 | emp24/gp25L/p24 family/GOLD [PF01105.19] |
| HYRNA1402_5493 | Basal disc | 4,80 | Alpha-L-arabinofuranosidase B (ABFB) [PF05270.8] |
| HYRNA1402_18715 | Basal disc | 4,79 | Galactose binding lectin domain [PF02140.13] |
| HYRNA1402_10822 | Basal disc | 4,78 | Peroxidase [PF00141.18] |
| HYRNA1402_13809 | Foot and bud | 4,78 | N.A. |
| HYRNA1402_15433.2 | Basal disc | 4,76 | Antistasin family [PF02822.9] |
| HYRNA1402_26258 | Basal disc and gastric column | 4,71 | Galactose binding lectin domain [PF02140.13] |
| HYRNA1402_32447 | Unspecific | 4,59 | N.A. |
| HYRNA1402_15641 | Unspecific | 4,33 | N.A. |
| HYRNA1402_10625 | Basal disc | 4,32 | Galactose binding lectin domain [PF02140.13] |
| HYRNA1402_25441 | Unspecific | 4,21 | N.A. |
| HYRNA1402_16547 | Basal disc | 4,20 | N.A. |
| HYRNA1402_16192 | Basal disc | 4,19 | Antistasin family [PF02822.9] |
| HYRNA1402_38642 | Basal disc | 4,13 | Galactose binding lectin domain [PF02140.13] |
| HYRNA1402_15433.3 | PCR failed | 4,13 | Antistasin family [PF02822.9] |
| HYRNA1402_9309 | Basal disc | 4,12 | Chitin binding Peritrophin-A domain [PF01607.19] |
| HYRNA1402_22117 | Unspecific | 4,00 | N.A. |
| HYRNA1402_26921 | Primer design failed | 3,92 | N.A. |
| HYRNA1402_14809 | Unspecific | 3,85 | PMP-22/EMP/MP20/Claudin family [PF00822.15] |
| HYRNA1402_30969 | Basal disc | 3,84 | N.A. |

**Continuation Table S2**

| HYRNA1402_15666 | Basal disc | 3,81 | Antistasin family [PF02822.9] |
| --- | --- | --- | --- |
| HYRNA1402_18002 | PCR failed | 3,76 | N.A. |
| HYRNA1402_45292 | Basal disc | 3,68 | N.A. |
| HYRNA1402_41082 | Basal disc | 3,66 | N.A. |
| HYRNA1402_37634 | Basal disc | 3,58 | N.A. |
| HYRNA1402_19926 | PCR failed | 3,52 | N.A. |
| HYRNA1402_9985 | Basal disc | 3,50 | N.A. |
| HYRNA1402_20745 | Gastric column | 3,31 | N.A. |
| HYRNA1402_17198 | Gastric column | 3,28 | Basic region leucine zipper  PF07716.10 |
| HYRNA1402_24690 | Basal disc | 3,27 | N.A. |
| HYRNA1402_24862 | Basal disc | 3,19 | Cystatin domain [PF00031.16] |
| HYRNA1402_23784 | Basal disc, gastric column and head | 3,13 | N.A. |
| HYRNA1402_15520 | Gastric column | 3,07 | N.A. |
| HYRNA1402_11567.2 | Basal disc and base of tentacles | 2,87 | FRG1-like family [PF06229.7] |
| HYRNA1402_11381 | Basal disc | 2,80 | Glutamine synthetase, catalytic domain [PF00120.19] |
| HYRNA1402_8319 | Unspecific | 2,70 | N.A. |
| HYRNA1402_33702 | Basal disc | 2,68 | N.A. |
| HYRNA1402_5816.1 | Gastric column | 2,58 | Sulfate transporter family  PF00916.15 |
| HYRNA1402_11655.1 | Unspecific | 2,39 | DOMON domain [PF03351.12] |
| HYRNA1402_19151 | Unspecific | 2,37 | N.A. |
| HYRNA1402_11408.2 | Unspecific | 2,30 | Glutamine synthetase, catalytic domain [PF00120.19] |
| HYRNA1402_14038.2 | Unspecific | 2,16 | Integral membrane protein DUF92 [PF01940.11] |
| HYRNA1402_20123.1 | Basal disc and endoderm whole body | 2,15 | Leucine Rich repeats (2 copies) [PF12799.2] |
| HYRNA1402_21745 | Basal disc | 2,00 | N.A. |
| HYRNA1402_7885 | Unspecific | 1,95 | Domain of unknown function (DUF3504) [PF12012.3] |
| HYRNA1402_26940 | Basal disc | 1,93 | Galactose binding lectin domain [PF02140.13] |
| HYRNA1402_18930 | Basal disc | 1,92 | N.A. |
| HYRNA1402_10653 | Basal disc | 1,91 | N.A. |
| HYRNA1402_26939 | Basal disc | 1,89 | Galactose binding lectin domain [PF02140.13] |
| HYRNA1402_23373 | Basal disc | 1,85 | Galactose binding lectin domain [PF02140.13] |
| HYRNA1402_24535 | Foot | 1,77 | PAN domain [PF00024.21] |
| HYRNA1402_11408.1 | Unspecific | 1,74 | Glutamine synthetase, catalytic domain [PF00120.19] |
| HYRNA1402_17607 | Unspecific | 1,69 | N.A. |
| HYRNA1402_1750.2 | Unspecific | 1,56 | ThiF family [PF00899.16] |
| HYRNA1402_16367 | Foot | 1,54 | N.A. |
| HYRNA1402_32349 | Unspecific | 1,53 | N.A. |
| HYRNA1402_3428 | Unspecific | 1,50 | Fibronectin type III domain [PF00041.16] |
| HYRNA1402_12395 | Unspecific | 1,45 | Fibronectin type III domain [PF00041.16] |
| HYRNA1402_23522 | Basal disc | 1,43 | N.A. |
| HYRNA1402_14079 | Basal disc | 1,40 | Fibronectin type III domain [PF00041.16] |

**Continuation Table S2**

| HYRNA1402_1801 | Unspecific | 1,28 | 7 transmembrane receptor (Secretin family) [PF00002.19] |
| --- | --- | --- | --- |
| HYRNA1402_23423 | Foot | 1,27 | N.A. |
| HYRNA1402_2635.2 | Unspecific | 1,19 | 3'5'-cyclic nucleotide phosphodiesterase [PF00233.14] |
| HYRNA1402_21369 | Foot | 1,13 | N.A. |
| HYRNA1402_315.2 | Unspecific | 1,10 | Dedicator of cytokinesis [PF06920.8] |
| HYRNA1402_15310 | Unspecific | 1,07 | DAN domain [PF03045.10] |
| HYRNA1402_17882.2 | Unspecific | 1,05 | MoaE protein [PF02391.12] |
| HYRNA1402_23194.2 | Unspecific | 1,04 | Death effector domain [PF01335.16] |
| HYRNA1402_4110.3 | Unsüpecific | 1,02 | N-acetyltransferase [PF00797.12] |
| HYRNA1402_13356 | Unspecific | 1,01 | Miro-like protein [PF08477.8] |
| HYRNA1402_16200 | Basal disc | 1,00 | N.A. |

**Table S3.** Anterior part-specific transcripts screened by in situ hybridization. The top-10 transcripts are shown below, an excel file with detailed information on all 2679 transcripts can be obtained from [marcelo.rodrigues@uibk.ac.at](mailto:marcelo.rodrigues@uibk.ac.at) or [peter.ladurner@uibk.ac.at](mailto:peter.ladurner@uibk.ac.at)

| **Transcript id** | **In situ Hybridization Pattern** | **LOG2 Fold Change** | **Protein Family [Pfam code]** |
| --- | --- | --- | --- |
| HYRNA1402_27622.1 | Gastric column | 8,90 |  |
| HYRNA1402_15485 | ISH failed | 7,69 | Astacin (Peptidase family M12A) [PF01400.19] |
| HYRNA1402_23485 | Head | 7,65 |  |
| HYRNA1402_8529 | Head | 7,47 | Astacin (Peptidase family M12A) [PF01400.19] |
| HYRNA1402_14924 | Head | 7,26 | Astacin (Peptidase family M12A) [PF01400.19] |
| HYRNA1402_17752 | PCR Failed | 6,80 |  |
| HYRNA1402_16561 | Head | 6,69 |  |
| HYRNA1402_14721 | Head | 6,62 | Astacin (Peptidase family M12A) [PF01400.19] |
| HYRNA1402_16617 | Head | 6,49 | Cysteine-rich secretory protein family [PF00188.21] |
| HYRNA1402_22404 | Head | 6,35 | Insulin growth factor-like family [PF14653.1] |

**Table S4.** Information about the 21 transcripts matching identified proteins from the nanoLC-ESI-MS/MS are shown below, an excel file with detailed information on all identified peptides can be obtained from [marcelo.rodrigues@uibk.ac.at](mailto:marcelo.rodrigues@uibk.ac.at) or [peter.ladurner@uibk.ac.at](mailto:peter.ladurner@uibk.ac.at)

| **Transcript ID** | **ΣCoverage** | **Σ# Proteins** | **Σ# Unique Peptides** | **Σ# Peptides** | **Σ# PSMs** |
| --- | --- | --- | --- | --- | --- |
| HYRNA1402_13087 | 39,07 | 1 | 11 | 11 | 42 |
| HYRNA1402_28063.1 | 34,19 | 3 | 4 | 4 | 20 |
| HYRNA1402_6988 | 34,00 | 1 | 18 | 19 | 60 |
| HYRNA1402_11464 | 34,94 | 1 | 11 | 12 | 32 |
| HYRNA1402_15963 | 28,67 | 2 | 6 | 7 | 18 |
| HYRNA1402_18991 | 18,37 | 1 | 4 | 4 | 9 |
| HYRNA1402_5493 | 47,03 | 1 | 29 | 30 | 95 |
| HYRNA1402_18715 | 55,02 | 1 | 10 | 10 | 31 |
| HYRNA1402_10822 | 43,35 | 2 | 14 | 16 | 46 |
| HYRNA1402_26258 | 56,02 | 2 | 6 | 9 | 48 |
| HYRNA1402_10625 | 57,47 | 1 | 17 | 19 | 97 |
| HYRNA1402_9309 | 2,45 | 1 | 1 | 1 | 3 |
| HYRNA1402_30969 | 82,48 | 1 | 4 | 9 | 41 |
| HYRNA1402_37634 | 82,88 | 1 | 8 | 8 | 24 |
| HYRNA1402_9985 | 58,80 | 1 | 21 | 24 | 80 |
| HYRNA1402_24690 | 78,09 | 1 | 13 | 15 | 103 |
| HYRNA1402_24862 | 20,79 | 1 | 3 | 3 | 9 |
| HYRNA1402_11567.2 | 45,48 | 1 | 4 | 13 | 48 |
| HYRNA1402_26940 | 50,31 | 1 | 3 | 5 | 25 |
| HYRNA1402_26939 | 50,31 | 1 | 3 | 5 | 32 |
| HYRNA1402_23373 | 57,37 | 1 | 8 | 8 | 32 |


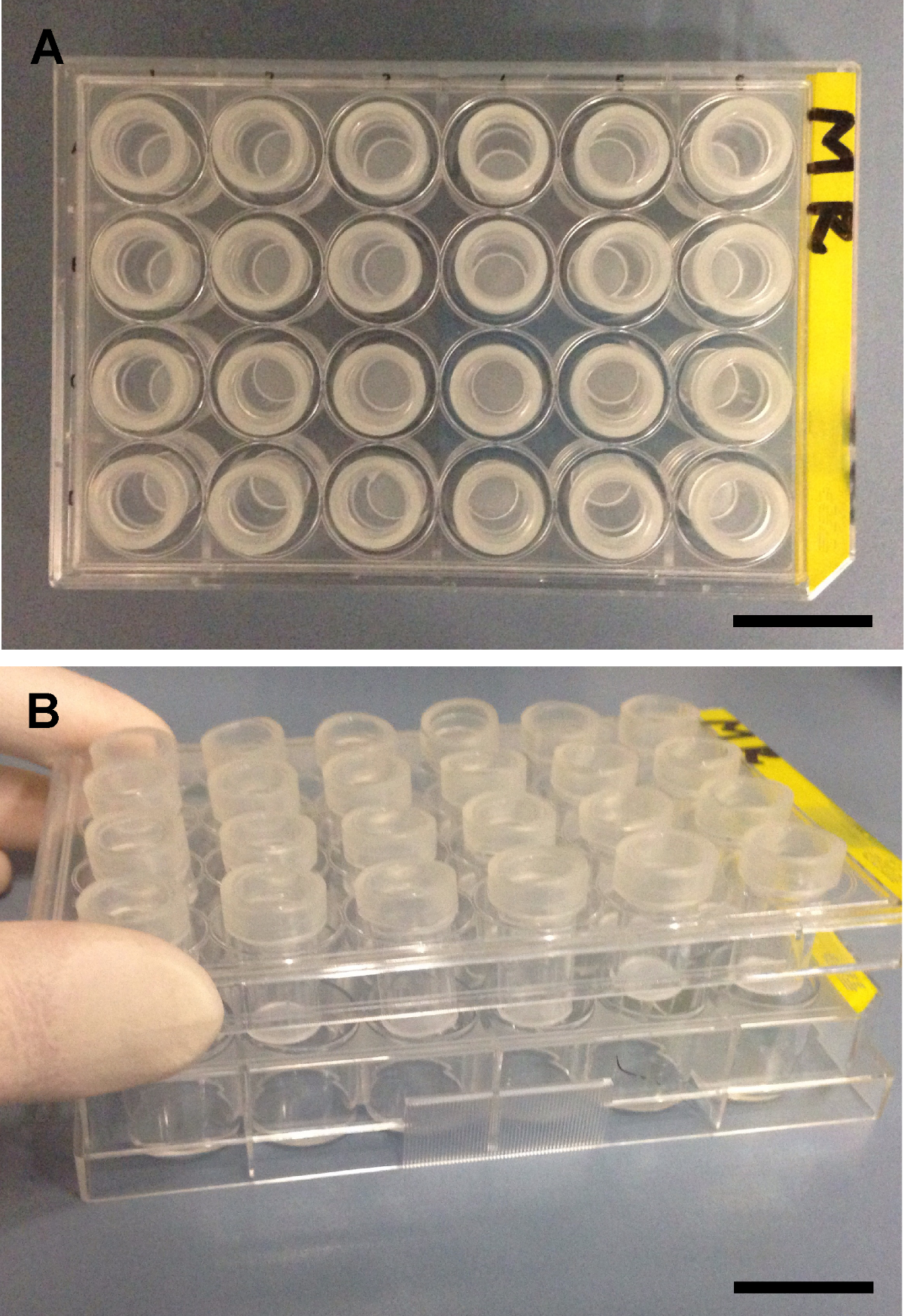


**Figure S1.** 24-well plate system used for *in situ* hybridization experiments. Scale bars: 2cm.


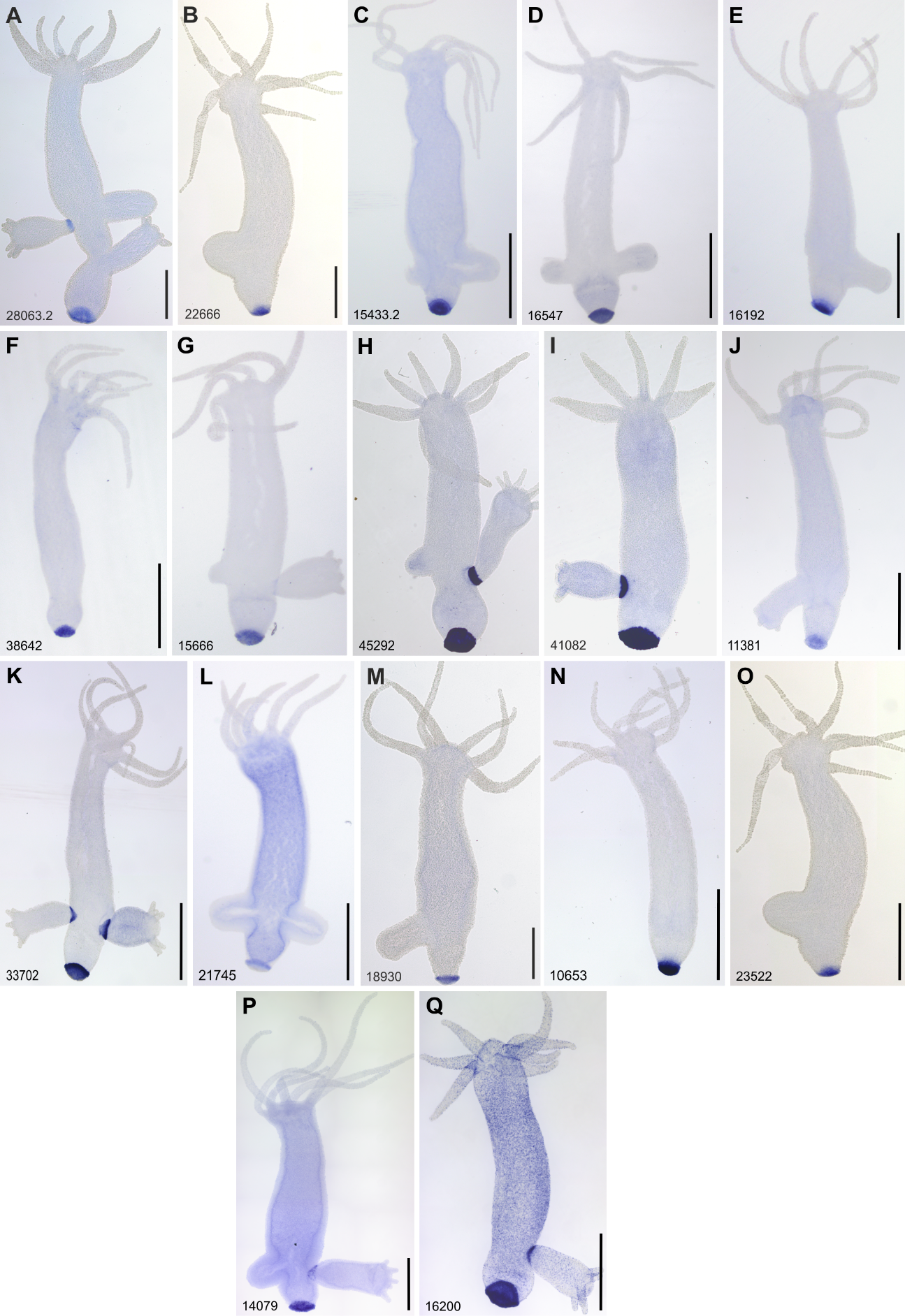
**Figure S2.** Whole mount in-situ hybridization of transcripts expressed in the basal disc but not found in the secretion. Scale bars: 500µm.


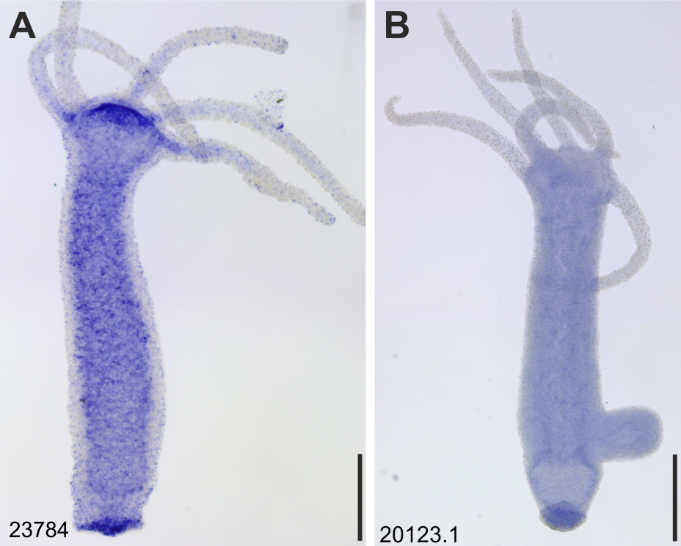


**Figure S3.** Whole mount in-situ hybridization of probes retrieving basal disc expression and additional expression in other cell lineage but not found in the secretion. Scale bars: 500µm.


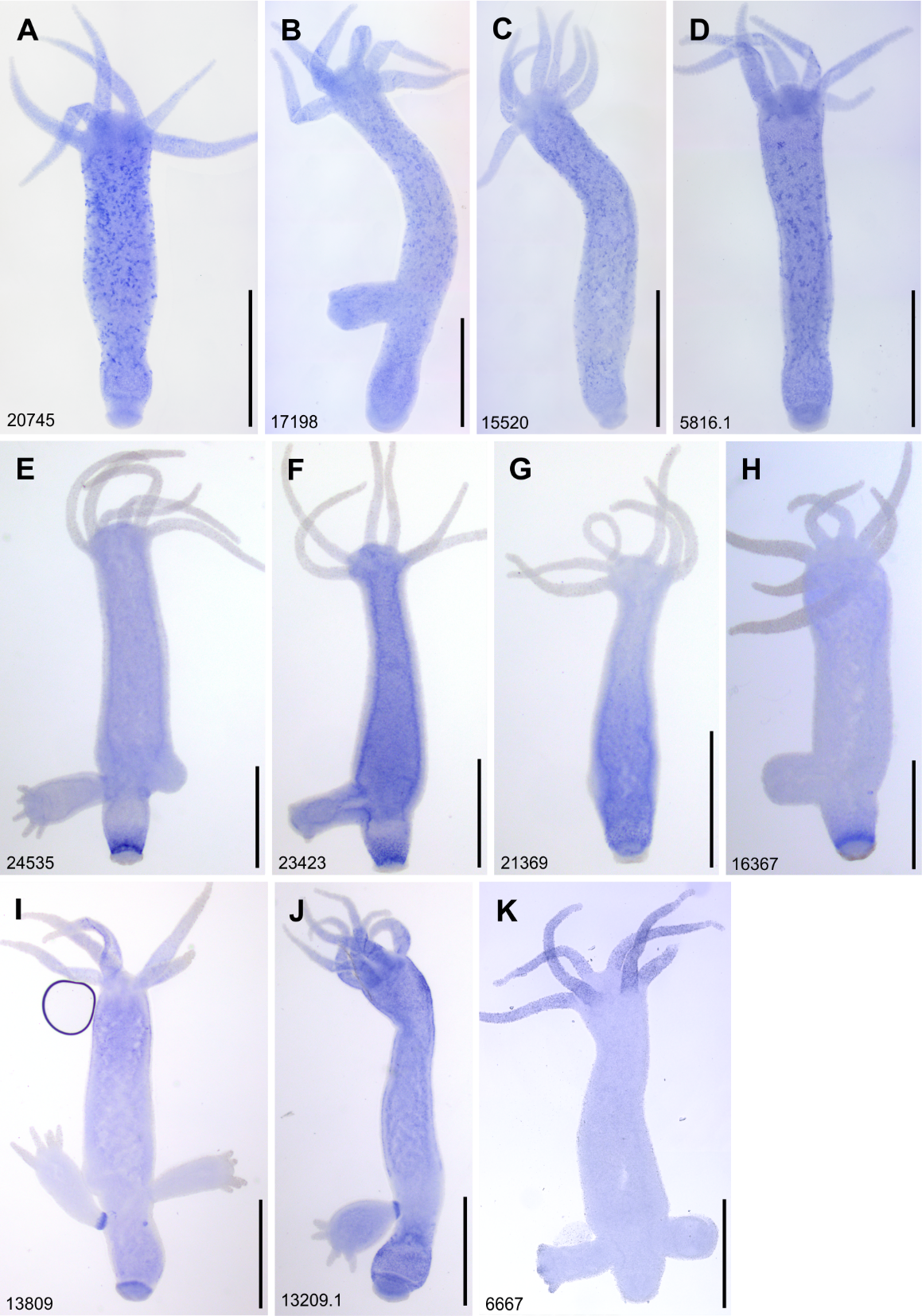


**Figure S4.** Whole mount *in situ* hybridization of probes present in the peduncle-specific list but not expressed in the basal disc. Scale bars: 500µm.


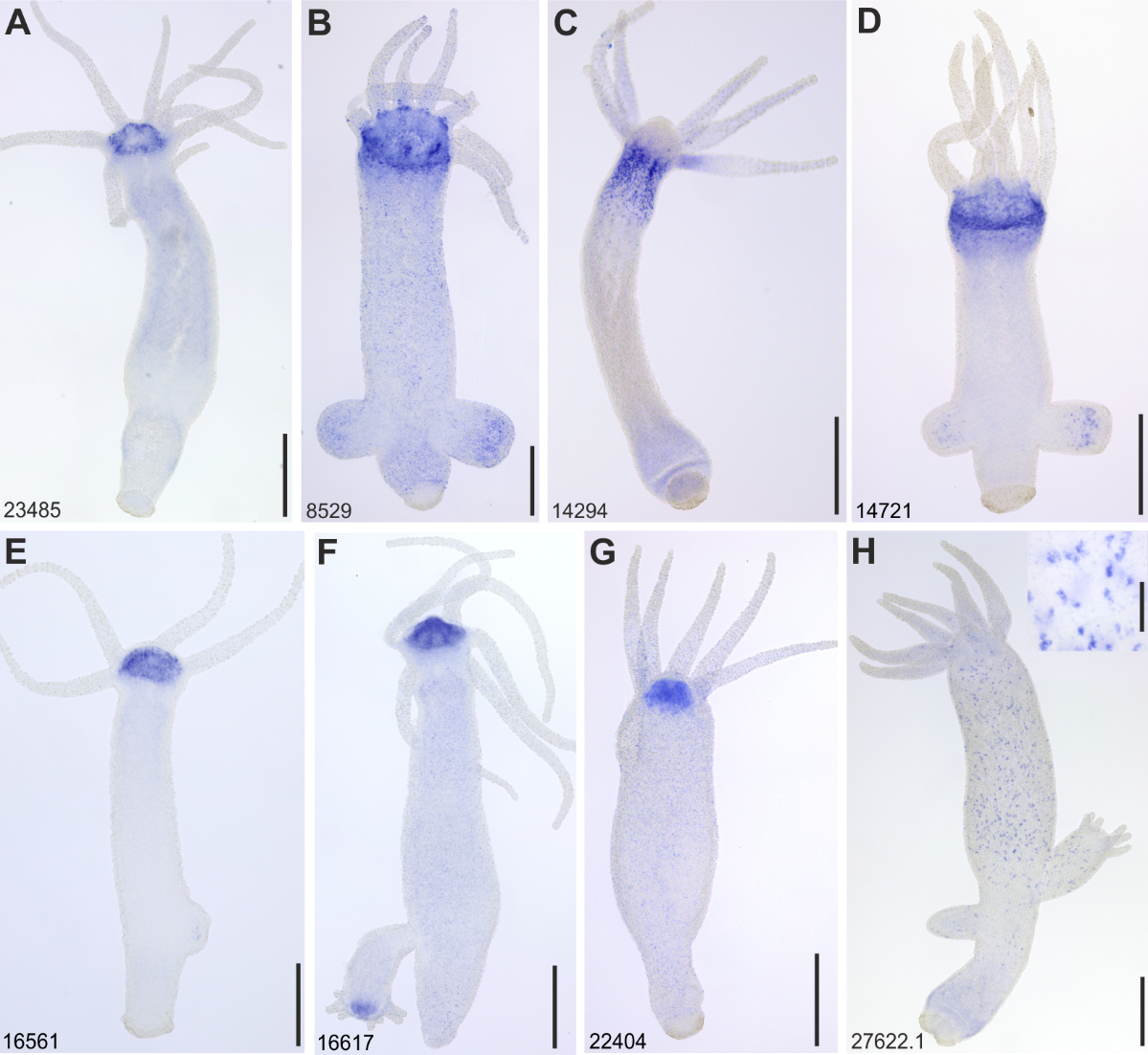


**Figure S5.** Whole mount *in situ* hybridization of representative anterior part-specific transcripts. Scale bars: 500µm, inset in figure H is 100µm.
